# Bifurcations and dynamics of a discrete predator–prey system

**DOI:** 10.1080/17513758.2014.927596

**Published:** 2014-06-17

**Authors:** Rasoul Asheghi

**Affiliations:** ^a^ Department of Mathematical Sciences, Isfahan University of Technology, Isfahan84156, Iran

**Keywords:** predator–prey system, Neimark–Sacker and period-doubling bifurcation, dynamics, host–parasite model, plant–herbivore model, 34K20, 92C50, 92D25

## Abstract

In this paper, we study the dynamics behaviour of a stratum of plant–herbivore which is modelled through the following *F*(*x, y*)=(*f*(*x, y*), *g*(*x, y*)) two-dimensional map with four parameters defined by

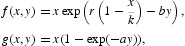

where *x*≥0, *y*≥0, and the real parameters *a, b, r, k* are all positive. We will focus on the case *a*≠*b*. We study the stability of fixed points and do the analysis of the period-doubling and the Neimark–Sacker bifurcations in a standard way.

## Introduction

The dynamics of a discrete-time mathematical model arises in a variety of applications especially in ecology and mathematical biology and is still a current topic of research. In ecology, predator–prey or plant–herbivore models can be formulated as discrete-time models. A plant–herbivore model (which is also of host–parasite type) applies to study the interaction between a plant species and a herbivore species. Discrete models described by difference equations for interacting populations are of considerable interest to biologists and agricultural ecologists. These models are actually more reasonable than the continuous-time models when populations have non-overlapping generations. This certainly happens for a population that has a one-year life cycle, such as insects. For example, gypsy moth larvae hatch from the egg mass after bud-break and feed on new leaves. At the end of the season, adult gypsy moths lay eggs and die. The gypsy moth is a notorious forest pest in North Central USA whose outbreaks are almost periodic and cause significant damage to the infested forests. A discrete-time model can exhibit more plentiful and hence more complicated dynamical behaviours than a continuous-time model of the same type. Jing and Yang [16] and Liu and Xiao [19] remarked that discrete-time prey–predator models have more complicated dynamics than those in continuous models.

In recent years, a significant number of published papers have been on mathematical models in biology. Mathematical models on prey–predator systems have created a major interest during the last few decades. Study of such systems with discrete- and continuous-time models can be found in [1,4,8,11–15,17,19].

Summers and colleagues [7] have analysed four typical discrete-time ecosystem models under the effects of periodic forcing. They observed that periodic forcing can produce a chaotic dynamics. Agiza *et al*. [2] found chaotic dynamics of a discrete prey–predator model with Holling's Type II response function. They did not consider the natural death rate of the predators. Kang and colleagues [4] observed quasi-periodicity, period-doubling and chaos in plant–herbivore interaction in the form of a host–parasite model. It is observed that the continuous model of such a system shows global stability of an interior equilibrium point.

Discrete-generation host–parasite models of the general form

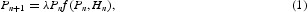

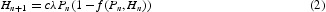

have been used to model the interaction between a host species (a plant) and a parasite species (a herbivore). In such models, *P*_*n*_ and *H*_*n*_ denote the population biomass of the host (a plant) and the parasite (a herbivore) in successive generations *n* and *n*+1, respectively. Here λ is the host's inherent rate of increase in the absence of the parasites, *c* is the biomass conversion constant and the function *f* represents the fraction of hosts surviving parasitism in each generation. Alternatively, *f* can be interpreted as the probability that each individual host escapes the parasites, so that the complementary term 1−*f* in the second equation is the probability of being parasitized.

The simplest version of this model is that of Nicholson–Bailey [5]:

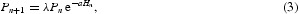

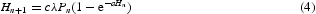

in which the proportion of hosts escaping parasitism is given by the Poisson distribution 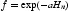, where *a* is the mean encounters per host. Thus, 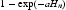 is the probability that a host will be attacked.

Beddington *et al*. [6] considered an extension of the Nicholson–Bailey host–parasite model:

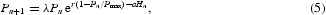

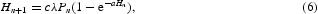

where *P*_max_ is the so-called environment-imposed ‘carrying capacity’ for the host in the absence of the parasite. The host density dependence is of the form 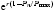. From Equations (5) and (6) we see that the host density dependence acts at a particular time in their life cycle in relation to the stage attacked by the parasites. The *H*_*n*_ herbivores search for *P*_*n*_ hosts before the density-dependent growth regulation takes effect. Hence, the next generation of herbivores depends on *P*_*n*_, the initial host population prior to parasitism.

In order to have a more realistic model, one can consider the system

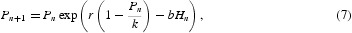

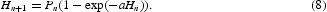

In the case *b*=*a*, this is the density-dependent predator–prey model studied by Beddington *et al*. [6]. By choosing an appropriate family chart (a parameter-dependent family of coordinate changes), one can assume *b*=*r*, and then the behaviour of the two populations just depends on the three positive parameters *a, r* and *k*. This system has the advantage that the dynamics restricted to 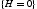 is given by the Ricker difference equation 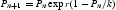, so the growth of the prey is limited and does not become unbounded.

Density-dependent models of the general form

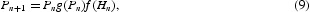

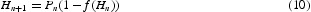

are considered in [3], where *f* denotes the fraction of prey surviving predation in each generation. For *b*≠*a*, system (7) and (8) is not of this type. We prove that this system can include both the features of the Neimark–Sacker bifurcation appearing in the system

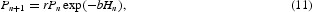

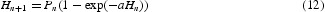

and the period-doubling bifurcations that are inherited from the Ricker map.

The existence of a Neimark–Sacker bifurcation in the model implies that both the host and parasite populations can oscillate around some mean values, and that these oscillations are stable and will continue indefinitely under suitable conditions.

It is proved in [12] that system (11) and (12) has a non-trivial steady-state solution which is stable for a certain range of parameter values, which is explicitly determined, and also undergoes a Neimark–Sacker bifurcation that produces an attracting invariant curve in some areas of the parameter space and a repelling one in others.

A general plant–herbivore model of the form

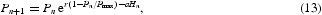

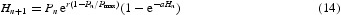

was considered in [4], making the following assumptions:

*Assumption 1* *P*_*n*_ represents the biomass of the plant population (nutritious) after the attacks by the herbivore but before its defoliation. *H*_*n*_ represents the biomass of the herbivore population before they die at the end of the season *n*.

*Assumption 2* Without the herbivore, the biomass of the plant population follows the dynamics of the Ricker model [21], namely

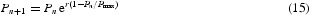

with a constant growth rate *r* and plant carrying capacity *P*_max_. The Ricker dynamics determines the amount of new leaves available for consumption for the herbivore.

*Assumption 3* It is supposed that the herbivores search for food randomly. The leaf area consumed is measured by the parameter *a*, i.e. *a* is a constant that correlates the total amount of the biomass that an herbivore consumes. The herbivore has a one-year life cycle, the larger the *a*, the faster is the feeding rate.

*Assumption 4* After attacks by herbivores, the biomass in the plant population is reduced to a fraction 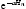 of that present in the absence of herbivores. Hence,

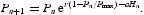



*Assumption 5* The amount of decreased biomass in the plants is converted to the biomass of the herbivore. It is assumed that the biomass conversion constant *c* is 1. Therefore, at the end of the season *n*, one has that

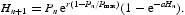



The bifurcation diagram in the parameter space of Equations (13) and (14) was presented by Armbruster *et al*. [4]. Two control strategies are suggested in [4]: reducing the population of the herbivore under some threshold and increasing the growth rate of the plant leaves.

Our paper is organized as follows. In Section 2, we give a brief discussion on the model under consideration. Fixed points and their stability are discussed in Section 3. Bifurcations analysis of the model is performed in Section 4.

## 2. Model

Consider the following 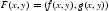 two-dimensional map with four parameters defined by

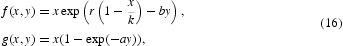

where *x*≥0, *y*≥0, and the real parameters *a, b, r, k* are all positive. Since we are interested in the case *a*≠*b*, we assume that *a*≠*b*. The corresponding recurrence equations are written as

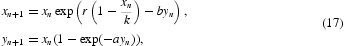

where *x*_*n*_≥0 and *y*_*n*_≥0. This is a generalized Beddington host–parasitoid model to study the interaction of certain plants and herbivores, where *x*_*n*_ and *y*_*n*_ stand for the density of two populations at time *n*, and *r* is the growth rate. In [10], Elaydi *et al*. investigate the stability and invariant manifolds of this model and also the stability of the coexistence fixed point. Elaydi *et al*. [10] obtain the stability region for positive fixed point in parameter space by using a numerical method.

In this paper, we study the stability of fixed points and do the analysis of the period-doubling and the Neimark–Sacker bifurcations in a standard way. Using an appropriate rescaling 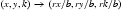, we can without loss of generality suppose that *b*=*r*>0 and *a*≠*r*. One can, of course, choose another family chart which permits to take *b*=1 and *a*≠1. For convenience, we prefer here to take *b*=*r* and *a*≠*r*. In that case, we can have at most three fixed points at 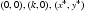, where 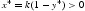 and y* is the unique positive solution of

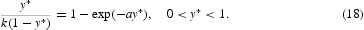

Also we have the following estimates on x*, y*:

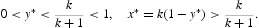



Let

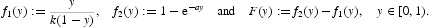



Then the interior equilibria are the intersection points of the functions *f*_1_ and *f*_2_ in the first quadrant. In addition, we have the following observations: *f*_1_(0)=0, 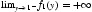, 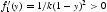; *f*_2_(0)=0, 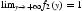, 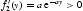; 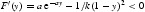 for *y*∈(0, 1) and *a*≤1/*k* while it has a unique zero at some *y*∈(0, 1) when *a*>1/*k*; and that

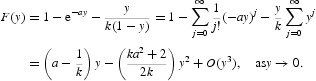



**Figure 1.**
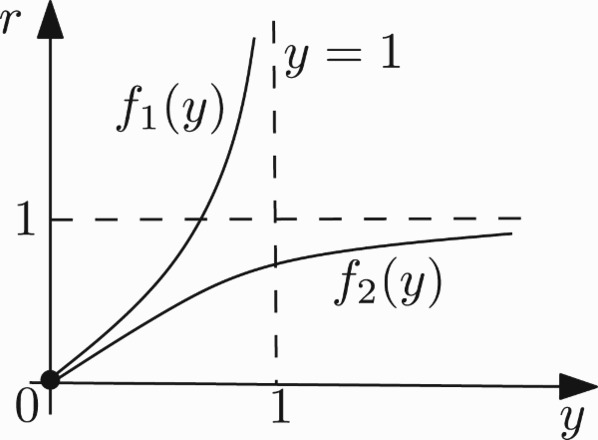
Graph of the functions *f*_1_ and *f*_2_ in the case 0<*a*≤1/*k*.

**Figure 2.**
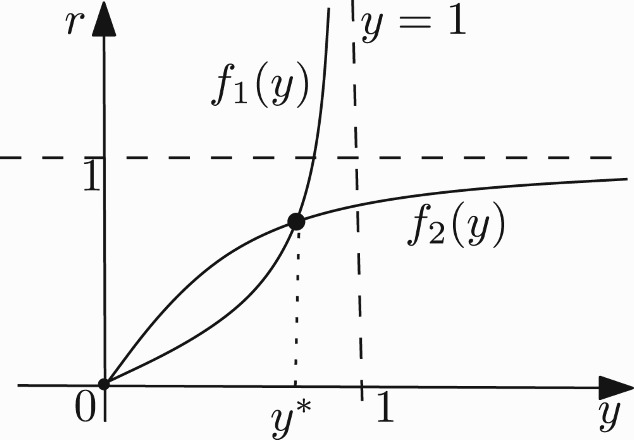
Graph of the functions *f*_1_ and *f*_2_ in the case *a*>1/*k*.

Thus if *ak*>1, then *F*(*y*)>0 for 0<*y*≪1 and if *ak*≤1, then *F*(*y*)<0 for 0<*y*≪1. Hence, the intersection of *f*_1_(*y*) and *f*_2_(*y*) in the first quadrant has 1 (Figure 1) or 0 (Figure 2) positive fixed point depending on the sign of *ak*−1. Therefore, we have the following propositions:

Proposition 2.1 For any *r*>0 and 0<*a*≤1/*k*, system (17) has no positive fixed points (Figure 1).

Proposition 2.2 For any *r*>0 and *a*>1/*k*, system (17) has a unique positive fixed point (Figure 2).

Now we turn to investigate the stability of system (17) by taking *b*=*r* and *a*≠*r*.

## 3. Stability of fixed points

In this section, we study the stability of fixed points via linearization, namely using the linear part of Equation (16) evaluated at each fixed point. To this end, we need the partial derivatives of *f* and *g*, which are as follows:

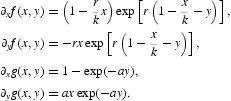



Since 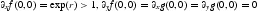, then the Jacobian matrix at the origin is

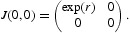



Hence, the extinction fixed point (0, 0) is a saddle point. This implies that plants cannot die out. It is noted that the lines 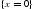 and 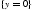 are invariant under the dynamics of Equation (16) such that 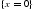 is the stable manifold of the origin (0, 0) and 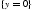 denotes the unstable manifold. The map restricted to 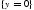 is the Ricker map given by

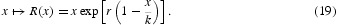

It is noted that the Schwartz derivative of the Ricker map *R*(*x*) defined in Equation (19) at *x*=*k* is calculated as follows:

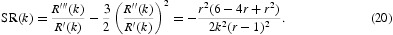

This derivative is negative provided that *r*≠1. Since for *r*=2 we have 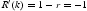 and 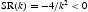, in this case, by the well-known arguments, any trajectory with the initial condition (*x*_0_, 0) on the *x*-axis with *x*_0_>0 goes towards the exclusion fixed point (*k*, 0). To obtain the Jacobian matrix at the exclusion fixed point (*k*, 0), we have that

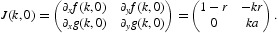



The eigenvalues of the above matrix are given by 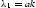 and 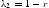. Depending on the location of the eigenvalues in the complex plane w.r.t. the unit circle, we have the following statements on (*k*, 0):

Proposition 3.1 The following hold.

1. If *ak*<1 and 0<*r*<2, then it is an attracting node.
2. If *ak*<1 and *r*>2, then it is a saddle point.
3. If *ak*>1 and *r*>2, then it is a repelling node.
4. If *ak*>1 and 0<*r*<2, then it is a saddle point.

*Proof* The proof is straightforward. For example, if *ak*<1 and 0<*r*<2, then 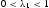 and 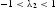; hence, we have a stable node at (*k*, 0).

Proposition 3.2 When *ak*=1 and 0<*r*<2, we have a non-hyperbolic fixed point at (*k*, 0), which is unstable.

*Proof* In the case *ak*=1 and 0<*r*<2, we compute below the centre manifold at (*k*, 0) and the dynamics restricted to the centre manifold to determine the orbit structure near (*k*, 0) in the first quadrant 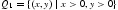. First, we bring the fixed point (*k*, 0) to the origin by using a linear translation, i.e. 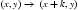. This yields the following 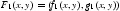 two-dimensional map with two parameters defined by

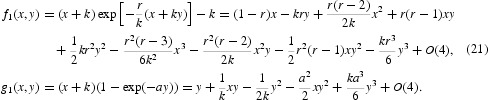

Next, we put the linear part of Equation (21) in Jordan normal form by using the linear transformation

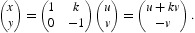

This induces the following two-dimensional map:

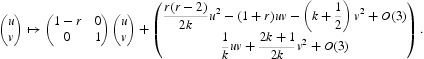

Thus, the centre manifold is given by the graph of

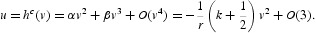

Then the dynamics on the centre manifold is given by the following scalar map:

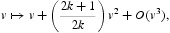

which shows that *v*=0 is unstable. If we return to the original coordinates, then the centre manifold at (*k*, 0) takes the form

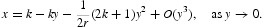

This centre manifold looks like a line near (*k*, 0).

Proposition 3.3 When *r*=2 and *ak*<1, the exclusion fixed point (*k*, 0) is asymptotically stable.

*Proof* Similar to the proof of Proposition 3.2, for the case *r*=2 and *ak*<1, we can get 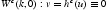 and

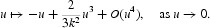



This shows that *u*=0 is asymptotically stable. This fact is consistent with Equation (20) for *r*=2.

*Remark 3.4* If either *ak*>1, *r*=2 or *ak*=1, *r*>2, then we have either 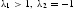 or 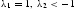, respectively. In consequence, there is a one-dimensional unstable direction and a one-dimensional centre direction at the point (*k*, 0). Therefore, this fixed point is unstable. Using the centre manifold theory, one can compute the centre manifold at this point.

*Remark 3.5* In the case *b*=*r*=2 and *a*=1/*k*, system (17) reduces to

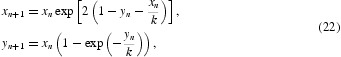

which has no positive fixed point. The extinction fixed point (0, 0) is a hyperbolic saddle with eigenvalues 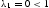 and 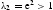. For this fixed point, the positive *y*-axis is the stable invariant manifold and the segment 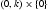 on the *x*-axis is the unstable invariant manifold. The exclusion fixed point (*k*, 0) is a non-hyperbolic fixed point with eigenvalues 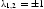. Numerical evidences show that any trajectory of Equation (22) with starting point contained in the first quadrant converges to the fixed point (*k*, 0) on the *x*-axis. But we were not able to prove analytically this claim. Consider now an arbitrary orbit 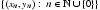 with initial condition (*x*_0_, *y*_0_) located in the first quadrant, namely with *x*_0_>0 and *y*_0_>0. First we observe that near the origin we have 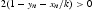, therefore 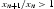, and hence 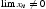. Now let us consider the functions *f*(*x*) and *g*(*y*) defined by

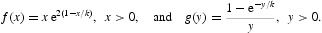



Because of having the estimates 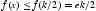 and *g*(*y*)≤1/*k*, we see that

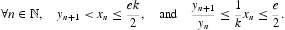

Here we write *e* for exp(1). As a result, after two iterations, our orbit lies entirely in the square 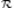 defined as 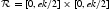.

In this way, we are finished with (*k*, 0). Now, we pay our attention to the positive fixed point (x*, y*) that exists for *ak*>1. The Jacobian matrix of Equation (16) at this fixed point is given by the following:

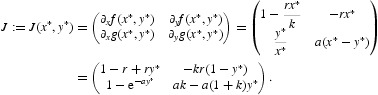



A direct computation implies that

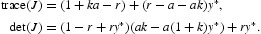



By the Jury test [9], we see that the positive fixed point is locally asymptotically stable if 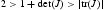. The biological implication of this inequality is simple: if it holds, then the plant–herbivore interaction exhibits simple stable steady-state dynamics. In domain 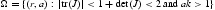 of the parameter space, we have that (x*, y*) is locally attractive. It follows from Equation (18) that

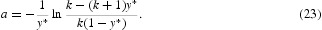

Since the function

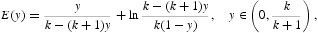

is strictly increasing on (0, *k*/(*k*+1)) and *E*(0)=0, then *E*(*y*)>*E*(0)=0 and hence

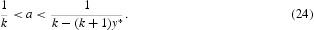

Under the assumption det(*J*)<1 and using Equation (23), we get

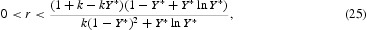

where

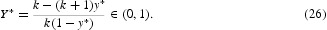

It is easily seen that

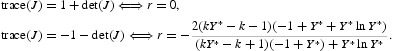



Let us define the following functions:

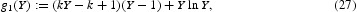

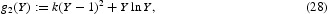

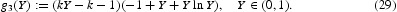

**Figure 3.**
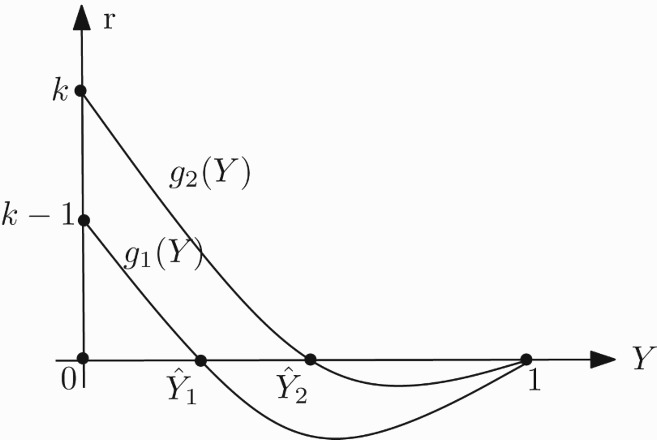
Graph of the functions *g*_1_ and *g*_2_ in the case *k*>1.

Then 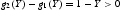 and for *k*>1 we have the following properties: 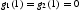; 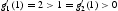; 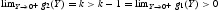. This implies that for *k*>1 and *Y*∈(0, 1), each one of the two functions *g*_1_ and *g*_2_ has a unique zero (Figure 3).

For *k*>1, let 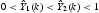 be the unique zeros of *g*_1_(*Y*) and *g*_2_(*Y*), respectively. Next we define

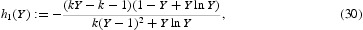

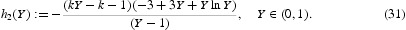

**Figure 4.**
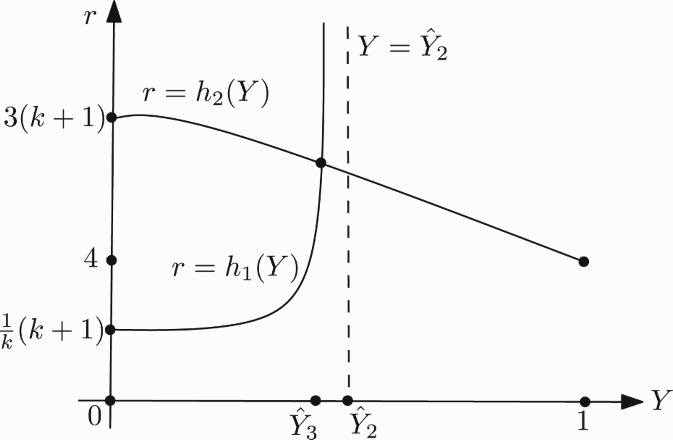
Position of *Ŷ*_3_(*k*) for *k*∈(⅓, 1].

Then, the unique solution 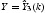 of the equation 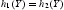 that exists for 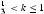, in which det(*J*)=1 and tr(*J*)=−2, lies in the interval 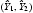 (Figure 4). Now, we can determine the basin of attraction Ω of (x*, y*), keeping *a*>1/*k* fixed, as

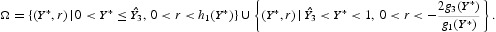

**Figure 5.**
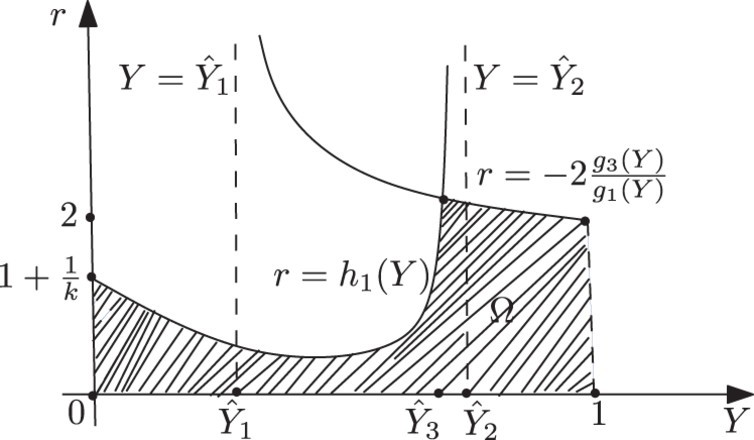
Shape of Ω in the case *k*>1.

The shape of the region Ω in (*Y, r*)-plane is shown in Figure 5. It is noted that Elaydi *et al*. [10] obtain the stability region for positive fixed point in parameter space by using a numerical method.

### 3.1. Case 0<*k*≤1

**Figure 6.**
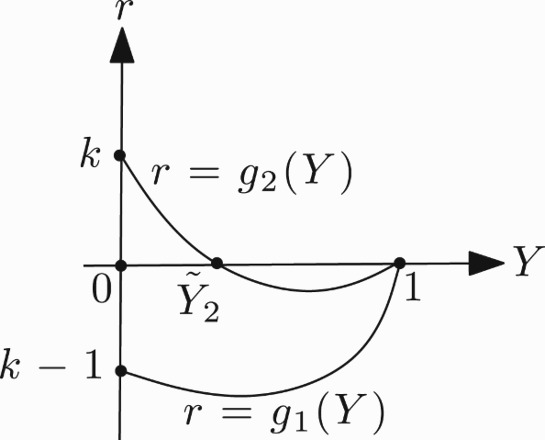
Graph of the functions *g*_1_ and *g*_2_ in the case 0<*k*≤1.

For the functions *g*_1_(*Y*) and *g*_2_(*Y*) defined in Equations (27) and (28), in this case, we have the following properties: 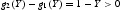 for *Y*<1; 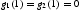; 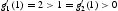; 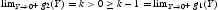. This implies that for 0<*k*≤1 and *Y*∈(0, 1) the function *g*_1_ has no zero, while *g*_2_ has a unique zero (Figure 6). For 0<*k*≤1, suppose that 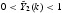 be the unique zero of *g*_2_(*Y*) and let *Ŷ*_3_(*k*) be as before, i.e. the unique solution of 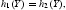 where 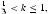 and the functions *h*_1_ and *h*_2_ are given by Equations (30) and (31). Then we have 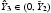. Therefore, the stability region Ω for the positive fixed point, in this case, is also determined by

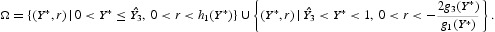

**Figure 7.**
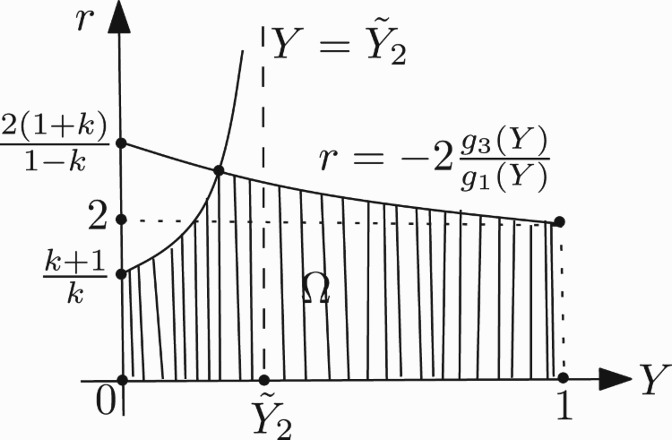
Shape of Ω in the case 0<*k*≤1.

For a picture of Ω in this case, see Figure 7.

It is worth noting that for a given *k*>0, when one chooses a pair (Y*, *r*) of Ω, the value of the parameter *a*>1/*k* is immediately specified by Equation (23).

Here, the local stability analysis of the fixed points is finished. Now we are in a position that our attention goes to treat bifurcations in the biological model under consideration. This will be done shortly in the next section.

## 4. Bifurcations of fixed points

In this section, we are planning to do the analysis of the local bifurcations of the fixed points of the system (16), including Neimark–Sacker and period-doubling bifurcations.

### 4.1. The Neimark–Sacker bifurcation

In the discrete setting, the Neimark–Sacker bifurcation is the analogue of the Hopf bifurcation that occurs in the continuous systems. It was discovered by Neimark [20], and independently by Sacker [22], who originally studied it in (connection) line with the stability of periodic solutions of ordinary differential equations, where it arises from the map obtained by taking a Poincare section transverse to the periodic flow. Hopf bifurcations create limit cycles in the phase plane of continuous models. On the other hand, Neimark–Sacker bifurcations generate dynamically invariant circles. As a result, we may find isolated periodic orbits as well as trajectories that cover the invariant circle densely. We seek conditions for Equation (16) to have a non-hyperbolic fixed point with a pair of complex conjugate eigenvalues of modulus 1. This happens surely at the interior fixed point (x*, y*). The associated Jacobian matrix 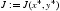 has two complex conjugate eigenvalues with modulus 1 in the case det(*J*)=1 and 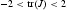. Hence, the candidate for the bifurcation curve is the curve

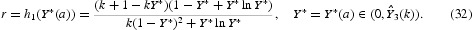

We consider (*k, a*) as fixed and take *r*>0 as a parameter and write it as *r*=r*+μ, where

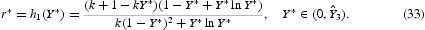

Following the standard way, we first must do some preliminary (linear or affine) transformations in order to put the linear part of the two-dimensional map (16) into normal form. This will be done shortly. Utilizing the linear transformation

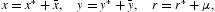

we get the following 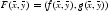 two-dimensional map defined by

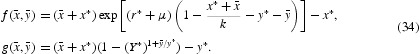

In the expanded form, we obtain that

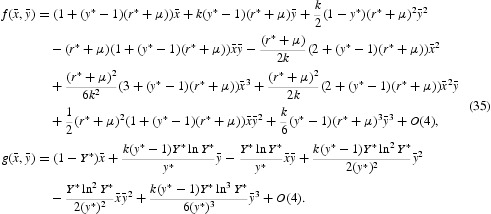

Now, the eigenvalues of the linear part of Equation (35) are given by

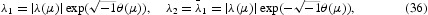

where 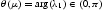. At μ=0 we have

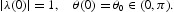



More precisely, it follows from Equation (35) that

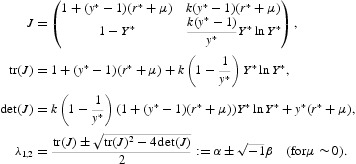



With a linear change of coordinates, we can put the two-dimensional map 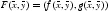 defined by Equation (34) in the following form:

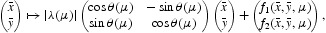

where 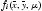, *i*=1, 2, are nonlinear in 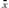 and 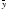. To simplify the notation, let us define

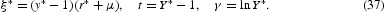

We make the linear transformation

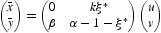

to obtain the following two-dimensional map defined by

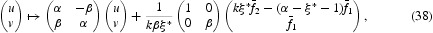

where

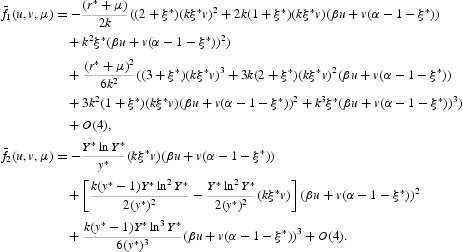

We can simplify Equation (38) further and arrive at

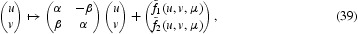

where

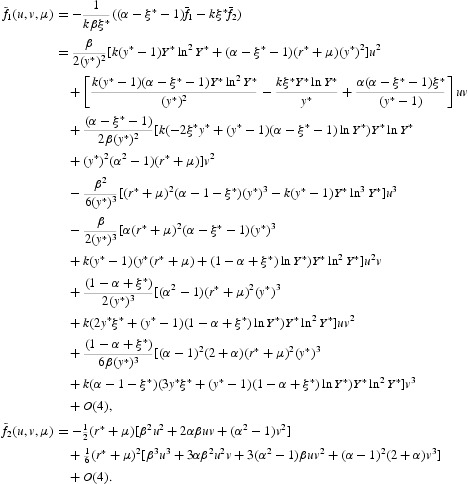



To analyse the corresponding bifurcation, we introduce the complex variables

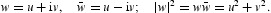



By Equation (39), the equation for *w* reads

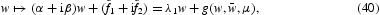

where

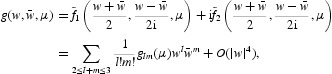

with

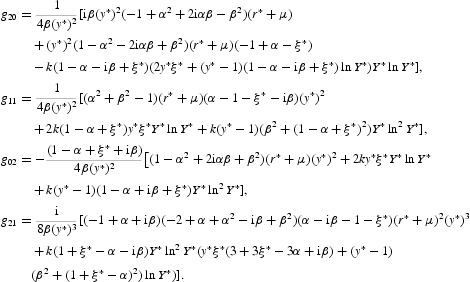



Now it follows from the normal form theorem for the Neimark–Sacker bifurcation that the one-dimensional map defined by Equation (40) can be transformed by an invertible parameter-dependent change of complex coordinate, which is smoothly dependent on the parameter,

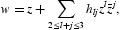

for μ near zero, into a map with only the resonant cubic term:

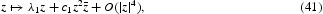

where

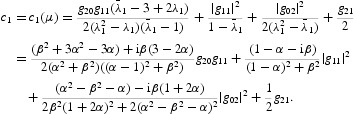



*Remark 4.1* Any such map (39) has the normal form (41) near μ=0 provided that 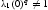 for *q*=1, 2, 3, 4. Subject to the further condition that 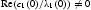, for sufficiently small μ the map has an invariant closed curve enclosing the origin when 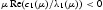. In the case that 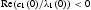, the bifurcation is said to be supercritical, and there is a stable attracting invariant curve for small enough μ>0, while a subcritical bifurcation arises for 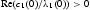, when there is a repelling invariant curve for small μ<0 (see [18] for more details).

Writing 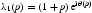 and 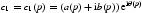, expression (41) can be written as

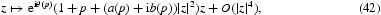

where

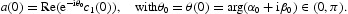

Using the representation 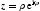, we obtain the following polar form of Equation (42):

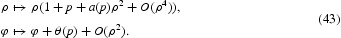

In the polar form (43), the ρ-map will depend on ϕ. If we neglect the higher order terms in Equation (43), then we will have the truncated polar form

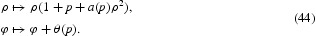

Bifurcations of the phase portrait of Equation (42) as *p* passes through zero can easily be analysed using the latter form, since the mapping for ρ is independent of ϕ. The first equation in Equation (44) defines a one-dimensional discrete dynamical system that has the fixed point ρ=0 for all values of *p*. The point is linearly stable if *p*<0; for *p*>0 the point becomes linearly unstable. The stability of the fixed point at *p*=0 is determined by the sign of the coefficient *a*(0). Suppose that *a*(0)<0; then the origin (ρ=0) is nonlinearly stable at *p*=0. Moreover, the ρ-map of Equation (44) has an additional stable fixed point

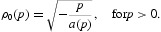

The ϕ-map of Equation (44) describes a rotation by an angle θ(*p*). Thus, by superposition of the mappings defined by Equation (44), we obtain the bifurcation diagram. The system always has a fixed point at the origin. This point is stable for *p*<0 and unstable for *p*>0. The invariant curves of the system near the origin look like the orbits near the stable focus of a continuous-time system for *p*<0 and like orbits near the unstable focus for *p*>0. At the critical parameter value *p*=0 the point is nonlinearly stable. The fixed point is surrounded for *p*>0 by an isolated closed invariant curve that is unique and stable. The curve is a circle of radius 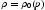. All orbits starting outside or inside the closed invariant curve, except at the origin, tend to the curve under iterations of Equation (44). This is a Neimark–Sacker bifurcation. The case *a*(0)>0 can be analysed in the same way. The system undergoes the Neimark–Sacker bifurcation at *p*=0. Contrary to the considered case, there is an unstable closed invariant curve that disappears when *p* crosses zero from negative to positive values. By Lemma 4.3 on page 128 (or Appendix 2) of [18], the higher order terms in Equation (43) do not affect the bifurcation of the closed invariant curve and in fact a locally unique invariant curve bifurcates from the origin in the same direction and with the same stability as in system (44). The coefficient *a*(0), which determines the direction of the appearance of the invariant curve in a generic system exhibiting the Neimark–Sacker bifurcation, can be computed via

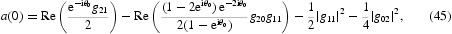

where 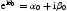. Since at μ=0 we have 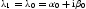, with

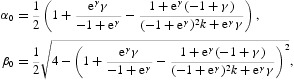

therefore

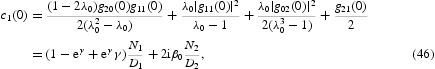

with

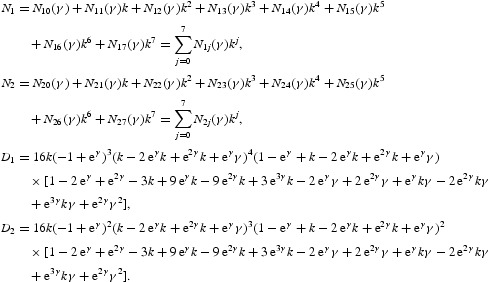

The formulae for computing the coefficients of *N*_1_ and *N*_2_ are omitted due to very long expressions. It follows from Equation (46) that

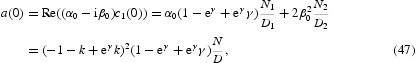

with

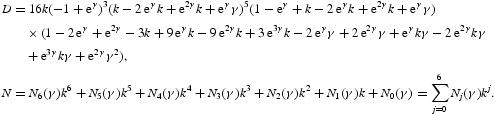

The formulas for specifying the coefficients of *N* are omitted due to very long expressions. Since

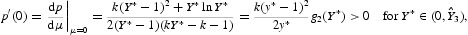

a unique closed invariant curve bifurcates from the positive fixed point when 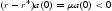. The sign of *a*(0) determines the stability of the invariant curve.

*Remark 4.2* Due to the technical nature of the coefficients (depending on the parameter) appeared for the expressions of *N*_1_, *N*_2_, *D*_1_, *D*_2_ in Equation (46) and for the expressions of *N, D* in Equation (47), we decided to choose some values for the parameters (*a, k*) and then follow the whole procedure constructed along the paper. By this choice of parameters, we can obtain the exact value of the positive fixed point (x*, y*).

**Figure 8.**
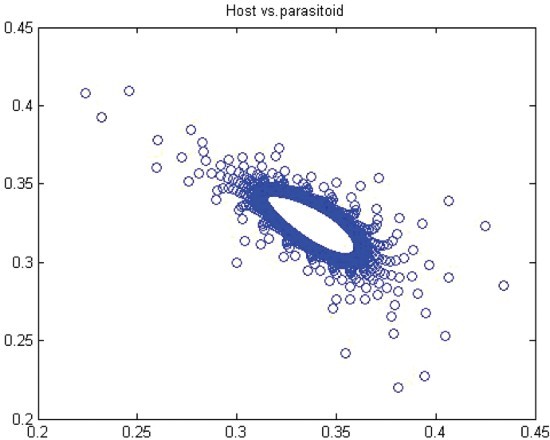
Phase diagram when *r*=3.7, *a*=10, *k*=0.5, (*x*_0_, *y*_0_)=(0.3, 0.3) and *n*=25, 000.

**Figure 9.**
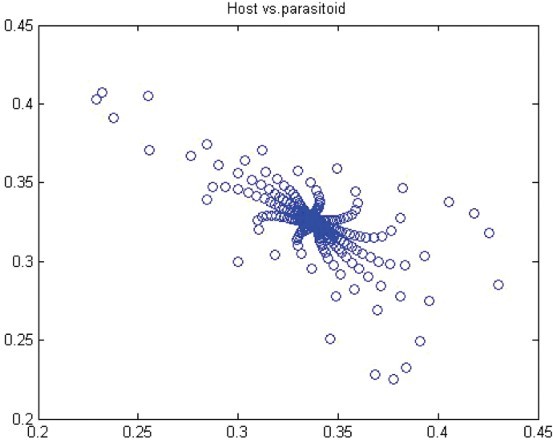
Phase diagram when *r*=3.6, *a*=10, *k*=0.5, (*x*_0_, *y*_0_)=(0.3, 0.3) and *n*=25, 000.

**Figure 10.**
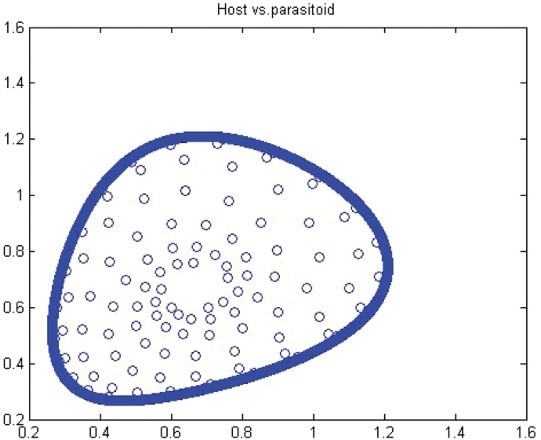
Phase diagram when *r*=1.6, *a*=27, *k*=2, (*x*_0_, *y*_0_)=(0.6, 0.6) and *n*=25, 000.

**Figure 11.**
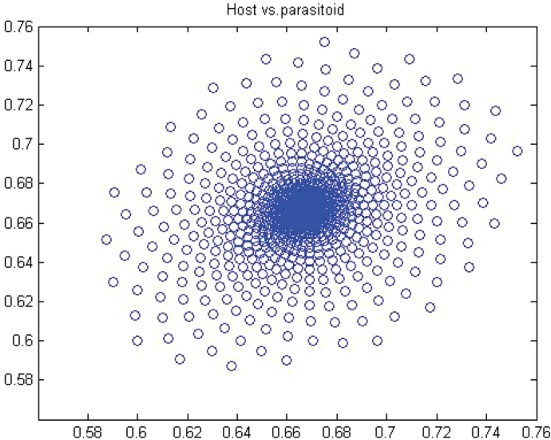
Phase diagram when *r*=1.49, *a*=27, *k*=2, (*x*_0_, *y*_0_)=(0.6, 0.6) and *n*=25, 000.

**Figure 12.**
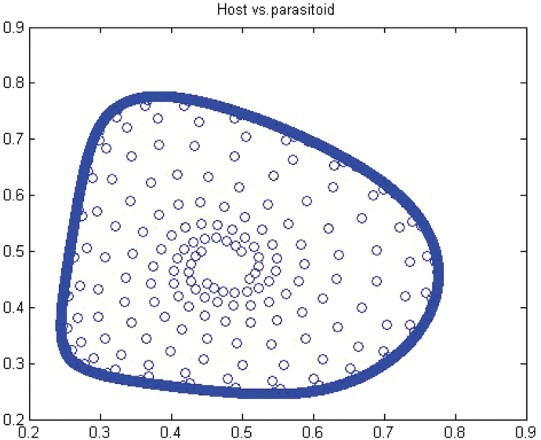
Phase diagram when *r*=2.2, *a*=50, *k*=0.9, (*x*_0_, *y*_0_)=(0.5, 0.5) and *n*=25, 000.

**Figure 13.**
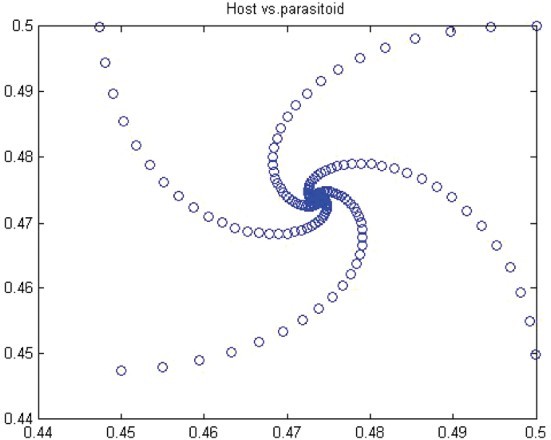
Phase diagram when *r*=2.0, *a*=50, *k*=0.9, (*x*_0_, *y*_0_)=(0.5, 0.5) and *n*=25, 000.

By following computations presented in the paper, we can finally find the value of *a*(0). For example, if we choose *a*=10 and *k*=0.5, then we find that 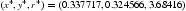 and then 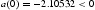. Thus, for *r*>r* we have a closed invariant curve which is stable (Figure 8). On this bases, Table 1 is constructed by using numerical computations. In order to support the results, phase diagrams for some particular parameter values would be presented for the Neimark–Sacker bifurcation in Figures 8–13.

**Table 1.** The numerical exact values for the positive fixed point (x*, y*) and for the coefficient *a*(0) corresponding to the chosen values of the parameters (*a, k*).

*k*	*a*	x*	y*	r*	θ_0_	*a*(0)	*Ŷ*_3_(*k*)
0.5	10	0.337717	0.324566	3.68416	2.31644	−2.10532	0.0787963
0.5	15	0.334101	0.331798	3.12737	2.12655	−0.572171	0.0787963
0.5	30	0.333338	0.333323	3.00145	2.09471	−0.156109	0.0787963
0.75	8	0.435051	0.419931	2.51322	1.74004	−0.341566	0.214038
0.75	5	0.456131	0.391825	3.45667	1.97182	−2.16554	0.214038
0.75	40	0.428571	0.428571	2.33333	1.73824	−0.0850811	0.214038
0.9	4	0.518854	0.423496	3.03818	1.75691	−1.29779	0.285252
0.9	7	0.482578	0.463803	2.20824	1.59711	−0.176965	0.285252
0.9	50	0.473684	0.473684	2.11111	1.59711	−0.0533712	0.285252
1	3	0.583714	0.416286	4.04332	2.0141	−4.87002	0.327559
1	13	0.500378	0.499622	2.00154	1.56665	−0.0657914	0.327559
1	23	0.500003	0.499997	2.00001	1.57074	−0.0625699	0.327559
2	3	0.741519	0.629241	1.31488	1.1322	0.000485813	0.588677
2	7	0.670936	0.664532	1.47064	1.29144	−0.00438717	0.588677
2	27	0.666667	0.666667	1.5	1.31812	−0.0351532	0.588677
20	10	0.952447	0.952378	1.04931	1.07542	−0.0152574	0.950933

### 4.2. Period-doubling bifurcation

In this subsection, we are planning to do the analysis of the period-doubling bifurcation. In the first step, let *k*>0 be fixed and consider the function

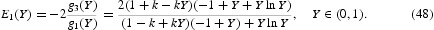

Then

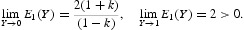



Moreover,

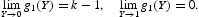



For *k*>1 we have *E*_1_(*Y*)>0 when 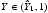, where *Ŷ*_1_ is the unique solution of *g*_1_(*Y*)=0. Clearly the values of *Ŷ*_1_ depend on 1<*k*<∞ adding that 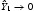 as 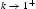, 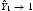 as 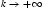, and *Ŷ*_1_=0.5 when *k*=3.38630. For 0<*k*≤1 the function *E*_1_(*Y*) is strictly decreasing and positive in the interval (0, 1). When *k*=1 this function is strictly decreasing from +∞ to +2.

We note that λ=1 is an eigenvalue of the Jacobian matrix *J* of the system (17) at the fixed point (x*, y*) if and only if 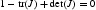 which yields *r*=0. Note that *r*=0 is not biologically of interest. On the other hand, λ=−1 is an eigenvalue of the Jacobian matrix *J* of the system (17) at the fixed point (x*, y*) if and only if 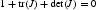 which yields *r*=*r*_1_, where 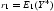 defined by Equation (48). The other eigenvalue of *J* for *r*=*r*_1_ is given by 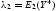, where

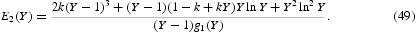

We have the following properties of the function *E*_2_(*Y*) defined by Equation (49):

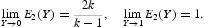



For 0<*k*<1, the function *E*_2_(*Y*) is strictly increasing on the interval (0, 1) and its values change from 2*k*/(*k*−1) to 1 when *Y* increases. When *k*>1, let 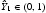 be the unique zero of *g*_1_(*Y*). Then one has for 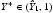 that 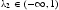. Now, we treat the dynamics on the centre manifold of the fixed point (x*, y*) in the case *r*=*r*_1_ with 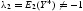. To do this, we have to place the fixed point at the origin. Let

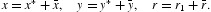



Then according to Equation (17) and using Equation (37), we get the following 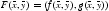 two-dimensional map defined by

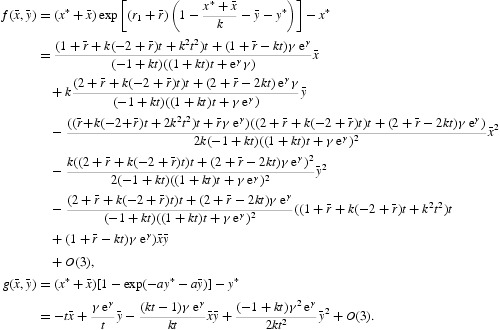



For 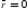 we have the eigenvalues 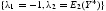 with eigenvectors

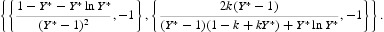



Introducing the change of variables

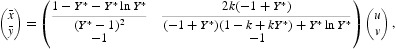

and applying to the previous map, we obtain the following two-dimensional map defined by

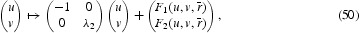

with

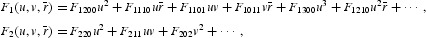

where the dots denote higher order terms, and with

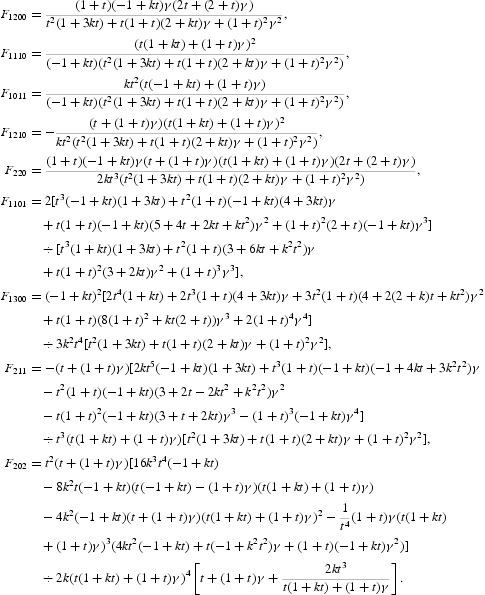



Here, we do not give the explicit expressions for the other coefficients of Equation (50), because they are out of use. In fact, the centre manifold will be given as a graph over *u* and 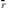, 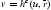, and be at least *O*(2). Thus, by computing the centre manifold as

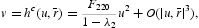

we can immediately see that the reduced map 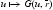 is given by

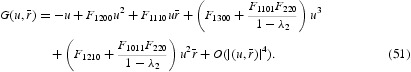

A direct calculation yields that

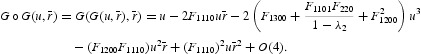



Therefore, the one-dimensional map 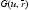 defined by Equation (51) undergoes a period-doubling bifurcation at 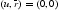 provided that

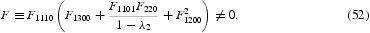

By an easy computation, we can find that

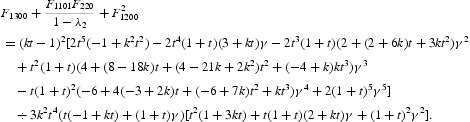



*Remark 4.3* Due to the complicated nature of the expressions (depending on the parameter) in Equations (50)–(52), we decided to choose some particular values for the parameters (*a, k*) and then follow the whole procedure made along this section. By this choice of parameters, we can determine the exact value of 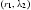. By following computations presented in this section, we can compute the value of *F* given by Equation (52). For this reason, Table 2 is constructed by using numerical computations.

**Table 2.** The numerical exact values for (*r*_1_, λ_2_, *F*) corresponding to the chosen parameter values for (*a, k*).

*k*	*a*	*r*_1_	λ_2_	*F*_1200_	*F*_1300_	*F*_1110_	*F*_1101_	*F*
5	0.5	12.0238	−2.62186	6.92079	−7.42265	0.191821	7.41292	6.30868
0.5	10	5.14677	−1.34479	4.77887	−7.38963	1.27529	5.00919	9.72169
0.75	5	6.4168	−1.58101	6.18323	−3.98477	0.708933	7.51087	15.4186
0.75	40	14	−5.99998	0.00001649	−0.725698	0.0285716	−13.9999	−0.020734
0.9	4	7.40901	−1.8899	5.1228	−2.06433	0.419045	4.88231	7.9262
0.9	30	37.9965	−17.9981	0.00017345	−0.172959	0.0030966	−37.9958	−0.0005355
1	3	6.52291	−1.30523	15.1958	−22.1696	1.5091	26.0496	99.6364
1	13	355.97	−176.11	0.0842284	0.392571	0.0000324	−352.337	0.000013
2	1	5.15839	−0.306219	−6.72308	33.6391	−1.01462	−16.2874	57.6695
5	0.4	6.45939	−0.619264	−16.2076	56.3775	−1.55496	−35.7933	199.463

### 4.3. Conclusions

In this paper, we studied a discrete-time predator–prey model which was a generalized Beddington–Nicholson–Bailey model. It is also a generalization of the system studied by Hone, Irle and Thurura. We investigated the stability and bifurcations of a generalized Beddington host–parasitoid model. We were able to compute the normal form coefficients of the Neimark–Sacker bifurcation without having the explicit form of the positive fixed point. The coefficients were very long and involved so that we decided to give numerical results along with phase diagrams (Tables 1 and 2 and Figures 8–13) to verify our findings. The presence of the Neimark–Sacker bifurcation was shown. The same process was done for the period-doubling bifurcation in standard way without knowing the exact value of the positive fixed point. For the positive fixed point, the stability region was obtained in (*Y, r*)-space. Moreover, the stability condition of the extinction fixed point (0, 0) and the exclusion fixed point (*k*, 0) was investigated in a standard way. Our system for values *b*=*r*=2 and *a*=1/*k* seems to have simple dynamics, but it needs further study to obtain the global dynamics. Future research will focus on the global stability of the system and on the study of the other types of bifurcations and dynamics phenomena. This includes a deeper discussion and a further study.
